# Adenovirus and Herpesvirus Diversity in Free-Ranging Great Apes in the Sangha Region of the Republic of Congo

**DOI:** 10.1371/journal.pone.0118543

**Published:** 2015-03-17

**Authors:** Tracie A. Seimon, Sarah H. Olson, Kerry Jo Lee, Gail Rosen, Alain Ondzie, Kenneth Cameron, Patricia Reed, Simon J. Anthony, Damien O. Joly, Denise McAloose, W. Ian Lipkin

**Affiliations:** 1 Zoological Health Program, Wildlife Conservation Society, Bronx, New York, United States of America; 2 Wildlife Health and Health Policy Program, Wildlife Conservation Society, Bronx, New York, United States of America; 3 Center for Infection and Immunity, Columbia University, New York, New York, United States of America; 4 Center for Sustainability and the Global Environment, University of Wisconsin, Madison, Wisconsin, United States of America; French National Centre for Scientific Research, FRANCE

## Abstract

Infectious diseases have caused die-offs in both free-ranging gorillas and chimpanzees. Understanding pathogen diversity and disease ecology is therefore critical for conserving these endangered animals. To determine viral diversity in free-ranging, non-habituated gorillas and chimpanzees in the Republic of Congo, genetic testing was performed on great-ape fecal samples collected near Odzala-Kokoua National Park. Samples were analyzed to determine ape species, identify individuals in the population, and to test for the presence of herpesviruses, adenoviruses, poxviruses, bocaviruses, flaviviruses, paramyxoviruses, coronaviruses, filoviruses, and simian immunodeficiency virus (SIV). We identified 19 DNA viruses representing two viral families, *Herpesviridae* and *Adenoviridae*, of which three herpesviruses had not been previously described. Co-detections of multiple herpesviruses and/or adenoviruses were present in both gorillas and chimpanzees. Cytomegalovirus (CMV) and lymphocryptovirus (LCV) were found primarily in the context of co-association with each other and adenoviruses. Using viral discovery curves for herpesviruses and adenoviruses, the total viral richness in the sample population of gorillas and chimpanzees was estimated to be a minimum of 23 viruses, corresponding to a detection rate of 83%. These findings represent the first description of DNA viral diversity in feces from free-ranging gorillas and chimpanzees in or near the Odzala-Kokoua National Park and form a basis for understanding the types of viruses circulating among great apes in this region.

## Introduction

With the exception of humans, all members of the family Hominidae, which includes a total of 6 species and 11 subspecies of chimpanzees, apes, and orangutans, are listed as endangered or critically endangered by the International Union for the Conservation of Nature (IUCN) (http://www.iucnredlist.org/). Human activities, notably hunting, habitat destruction and degradation from mining, agriculture and other extractive practices, are the most significant historical and ongoing factors driving population declines and local extinctions. Infectious disease, in particular Ebola virus disease, has more recently been recognized as an additional significant conservation threat that has caused periodic outbreaks with mortality as high as 90% in some populations [1].

Overlap of human and non-human great ape habitats provides opportunity for transmission of pathogens between these closely related species. Approximately 60% of emerging infectious diseases in humans are zoonotic (have a non-human origin) and of these, approximately 70% come from wildlife [2,3]. Malaria, Ebola, and HIV are among the most globally important and economically challenging infectious diseases in humans; all had their origin in a non-human primate host [4–8]. Conversely, wildlife also face challenges through increased contact and subsequent disease transmission from humans, as has been documented in outbreaks and mortality due to polio in chimpanzees and respiratory tract diseases, including influenza and metapneumovirus infections, in chimpanzees and gorillas [9–11].

Despite a developing understanding of the ecology and significance of pathogens and the diseases they cause in primates, it is estimated that only half of all micro- and macro-organisms have been identified even in the most well-studied non-human primate species [12]. This gap in our understanding of both the normal microbial flora and presence and significance of pathogens in non-human great apes has relevance both for these animals and humans, as intraspecies and zoonotic or zooanthroponotic transmission can have devastating effects in naïve populations in either group [13,14].

Here we report the first broad pathogen analysis of fecal samples obtained from free-ranging central chimpanzees (*Pan troglodytes troglodytes*) and western lowland gorillas (*Gorilla gorilla gorilla*) from within or bordering Odzala-Kokoua National Park (OKNP), Republic of Congo; an area containing significant numbers and the highest density of these two species of great apes in Central Africa [7,15].

## Materials and Methods

### Ethics statement

Fecal samples from free-ranging wild western lowland gorillas (*Gorilla gorilla gorilla*) and central chimpanzees (*Pan troglodytes troglodytes*) were collected from the environment. Collections were conducted by permission of the Congolese Ministry of Scientific Research (permit Nos. 003/MRS/DGRST/DMAST and No. 014/MRS/DGRST/DMAST) and in accordance with the American Society of Primatologists' Principles for the Ethical Treatment of Non-human Primates: (https://www.asp.org/society/resolutions/EthicalTreatmentOfNonHumanPrimates.cfm). The non-invasive nature of sample collection negated the need for further ethical review.

### Sample collection and storage

A total of 35 reconnaissance walk surveys for fecal collection were conducted between 2006 and 2010 in 13 locations in (n = 1) or to the east (n = 12) of the OKNP, and feces were collected from 12 locations. National Route 2 is a major north-south road that services several populated human communities and bisects the western portion of the study zone. The areas surveyed represent varying levels of shared habitat use by humans and non-human primates. Species of origin for each fecal sample was provisionally determined by morphological analysis of feces using previously described methods [16]. Feces were preserved in RNAlater and held at ambient temperature for 130 to 1,679 days (mean storage duration = 796 days) prior to shipment from the Republic of Congo to the United States for diagnostic testing. All appropriate export and import permits were received prior to shipment. Upon arrival in the US, samples were stored at −80C until processed.

### Viral testing

Total nucleic acid was extracted (QIAamp DNA stool mini kit; Qiagen Inc.; Valencia CA, USA) from 0.5 mL packed volume of pelleted feces and quantified (Nanodrop ND-1000 Spectrophotometer; Nanodrop Technologies; Wilmington, DE, USA) for all samples and RNA quality (Agilent Technologies Bioanalyzer; Santa Clara, CA, USA) was determined for a subset. PCR amplification was performed using one or more sets of primers (Eurofins MWG Operon; Huntsville, AL, USA) for each target pathogen and the Qiagen One-Step RT-PCR kit for RNA viruses (Qiagen Inc.; Valencia, CA, USA) or Amplitaq 360 Mastermix for DNA viruses (Life Technologies; Grand Island, NY, USA). Fecal samples were screened for the following genes and viruses using consensus and/or nested PCR as described elsewhere: DNA polymerase or glycoprotein B for herpesviruses [17–20], DNA polymerase for adenoviruses [21], L gene for filoviruses [22,23], L gene (large polymerase) for paramyxoviruses [23], RNA-dependent RNA polymerase for coronaviruses [24,25], NS5 gene for flavivirus [26], polymerase for orthopoxviruses [27], NS1 gene for bocavirus [28], and polymerase for SIV [29,30]. Amplified PCR products of appropriate molecular weight were purified using ExoSAP-IT (Affymetrix; Santa Clara, CA, USA) or gel purified (Qiagen MinElute Gel Extraction Kit; Qiagen Inc.; Valencia, CA, USA). Products were directly sequenced or subcloned into plasmid vectors (TOPO TA cloning kit; Life Technologies; Grand Island, NY, USA) and sequenced in the forward and reverse directions using an ABI 3730x/DNA analyzer for capillary electrophoresis and fluorescent dye terminator detection (Genewiz Inc., South Plainfield, NJ, USA)

### DNA barcoding and microsatellite analysis

Samples were genotyped through PCR amplification of a 450–500 basepair (bp) D loop region of mitochondrial DNA [31]. In some samples, an additional 386 bp portion of the mitochondrial 12S gene was used to confirm the species [32]. PCR products were directly sequencing (Genewiz; South Plainfield, NJ) and DNA was analyzed using Geneious (v.5.6.3 software created by Biomatters; available from http://www.geneious.com). To control for resampling from the same individual, microsatellite analysis through capillary electrophoresis was performed using the following 8 loci: D18s536, D4s243, D10s676, D9s922, D2S1326, D2S1333, D4S1627, and D9S905. Microsatellite loci were analyzed in Geneious and compared in Cervus 3.0 (developed by Field Genetics, London, UK). Samples identified from the same individual were tested for viruses individually and the combined viral PCR results were pooled as the representative for the diversity within that individual.

### Phylogenetic analysis

Nucleotide sequences were trimmed and aligned against each other and with available sequences in GenBank (GenBank, National Center for Biotechnology Information. http://www.ncbi.nlm.nih.gov) using the Geneious alignment tool (Geneious Pro 5.1.7 software; Biomatters LTD. Auckland, NZ) and Bayesian analysis was performed (MrBayes 3.1 plugin in Geneious Pro using gamma distributed rate variation using a general HKY85 substitution model [21]). The first 25% of a 1,100,000 chain length was discarded as burn-in, and four heated chains were run with a sub-sampling frequency of 200 [33]. Trees were finalized and labeled (FigTree v1.3.1 software, 2006–2009, Andrew Rambaut; Institute of Evolutionary Biology, University of Edinburgh. http://tree.bio.ed.ac.uk/). For adenovirus analysis, DNA sequences were analyzed by BLASTn and binned into subgroups based on sequence homology of greater than 98% nucleotide identity with closely related simian and human strains in GenBank. Sub-groups of simian adenoviruses were assigned to denote sequences that could not be phylogenetically separated from closely related strains because they were >99% identical to two or more simian adenoviruses.

### Statistical analysis

Binomial logistic regression was performed in R (version 3.0.2, 2014) to determine significant statistical differences in virus prevalence between chimpanzees and gorillas that adjusted for storage conditions using a z-score of ambient storage days [34]. When significant (p<0.05), odds ratios were calculated. Similar to Anthony et al, 2013 [35], rarefaction curves, nonparametric viral richness estimates (Chao2, Jack1 and ICE), and the Chao2 95% confidence interval based on 50 randomizations with R packages vegan and fossil were also performed. Univariate linear regression was used to describe the relationship of virus discovery with ambient storage duration.

## Results

**Fig 1:**
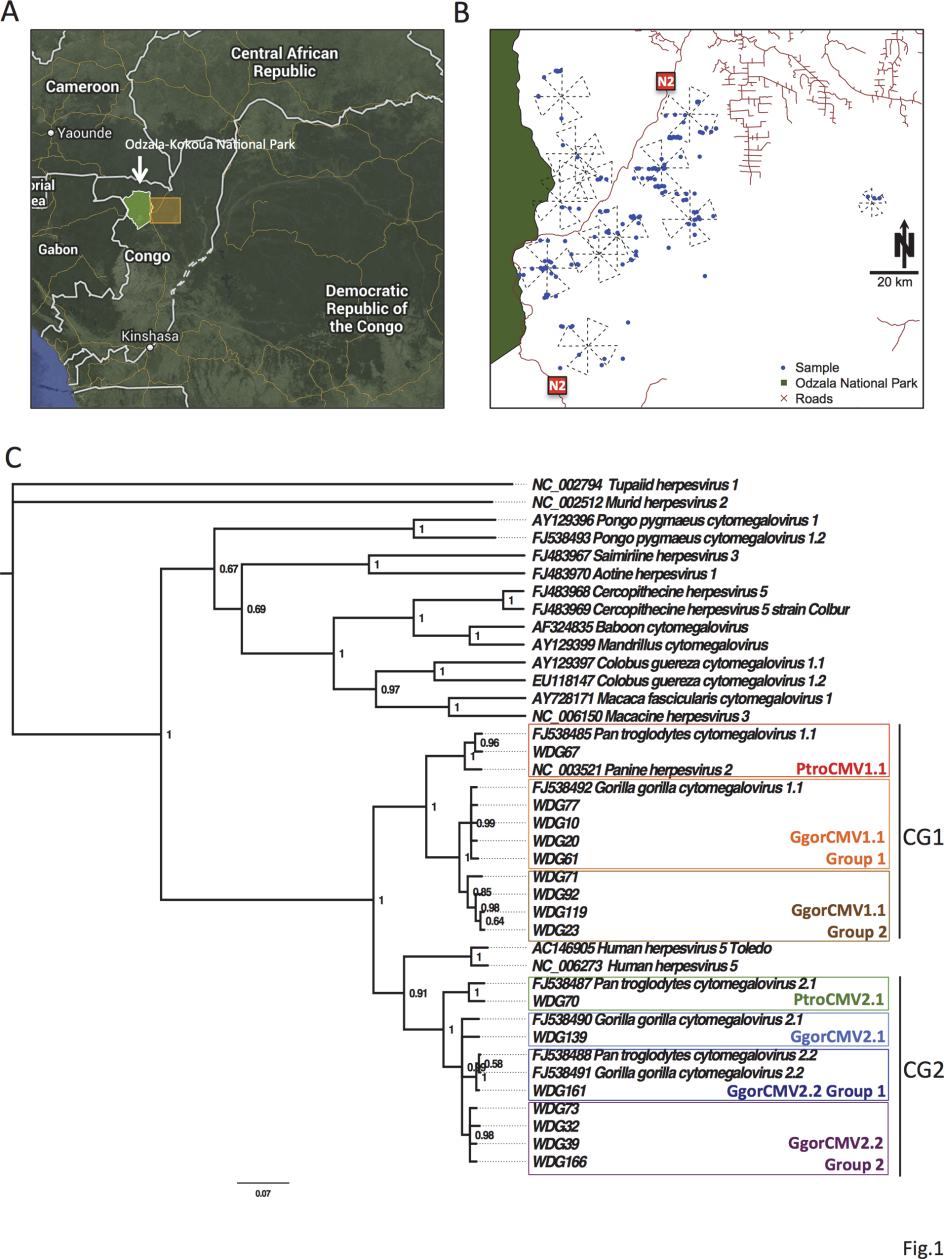
Location of sites and phylogenetic tree of herpesvirus lineages found in gorillas and chimpanzees. A. Map of the Republic of Congo showing the location of Odzala-Kokoua National Park (arrow) and the study area (yellow box). B. Shows the 12 sites where the samples were collected. Blue dots show where each sample was collected and the crosses show the extent of the survey sites. C. Phylogenetic tree of betaherpesviruses found in gorillas and chimpanzees. The tree was constructed through a nucleotide alignment of the herpesvirus CMV glycoprotein B gene using Bayesian analysis. Sample identification (WDG number) and Genbank accession numbers are shown. Bayesian posterior probabilities of branching demonstrate the robustness of the individual groups. *Tupaiid herpesvirus 1* was used as the outgroup.

Fecal samples were collected between 2006 and 2010 from 12 of the 13 sites east of OKNP (Fig. 1). Feces were identified to species, based both on morphological characteristics and genetic analysis of mitochondrial D loop gene. Individual apes were identified through microsatellite markers. Great ape species was confirmed in all of the samples. Four of 166 samples were of human origin and were eliminated from the study. In the remaining 162 samples, duplicates were received from 3 individuals, for a total sample population of 159 individuals. Morphologic assignment to ape species, which was confirmed by genetic testing, was correct for 147 individuals (92.5%). Genetic testing confirmed sample collection from 136 (85.5%) western lowland gorillas and 23 central chimpanzees.

Fecal samples were tested for the presence of herpesvirus, adenovirus, poxvirus, and bocavirus DNA and flavivirus, filovirus, paramyxovirus, coronavirus, and SIV RNA by PCR. Only herpesviruses and adenoviruses (Table 1 and S1 Table) were detected. BLASTn analysis results are shown in S2 Table. Overall, 57.9% (92 of 159) individual primates were positive for one or more virus; 31.4% (50 of 159) were positive for two or more viruses. Twenty-six samples contained more than one adenovirus, 19 contained more than one herpesvirus, and 27 contained both adenoviruses and herpesviruses (S1 Table). No RNA viruses and no viruses of known human origin were detected. Failure to detect RNA viruses may reflect degradation due to suboptimal long-term storage in RNALater, as samples were stored for extended periods at ambient temperature rather than frozen; the latter being the vendor recommendation for long term (>4 weeks) storage. Indeed, when tested, RNA concentrations were variable and often at low levels, ranging from 3–300 ng/μL in the undiluted total nucleic acid extracts. No intact 18S or 28S rRNA was detected by Bioanalyzer analysis. Accordingly, we focus in this report on the DNA viruses.

**Table 1:** Virus prevalence in chimpanzees and gorillas.

				Gorilla (n = 136)		Chimpanzee(n = 23)	
Virus family or subfamily	Genus	Species (abbr.)	Abbr. Virus or group	Positives	Prevalence (%)	Positives	Prevalence (%)
*Herpesviridae*				32	23.5	9	39.1
*Gammaherpesvirinae*				30	22.1	9	39.1
	*Lymphocryptovirus*	GoHV-1	GgorLCV1	29	21.3	3	13.0
		*	GgorLCV2	2	1.5	0	0.0
		*	PtroLCV1	0	0.0	6	26.1
	*Rhadinovirus*	*	GgorRHV1	1	0.7	0	0.0
		*	PtroRHV	0	0.0	1	4.3
*Betaherpesvirinae*	*Cytomegalovirus*			17	12.5	2	8.7
		*	GgorCMV1.1 Group 1	5	3.7	0	0.0
		*	GgorCMV1.1 Group 2	3	2.2	0	0.0
		*	GgorCMV2.1	5	3.7	0	0.0
		*	GgorCMV2.2 Group 1	2	1.5	0	0.0
		*	GgorCMV2.2 Group 2	4	2.9	0	0.0
		*	PtroCMV1.1	0	0.0	1	4.3
		*	PtroCMV2.1	0	0.0	1	4.3
*Adenoviridae*				61	44.9	16	69.6
	*Mastadenovirus*	HAdV-B	SAdVGroupOKNP	30	22.1	2	8.7
		HAdV-B	SAdVGroup27.1/28.2/29/46/47	26	19.1	1	4.3
		HAdV-B	SAdVGroup27.2/28.1/32/41.1/41.2	6	4.4	0	0.0
		HAdV-B	SAdVGroup35.1/35.2	2	1.5	1	4.3
		HAdV-C	SAdV31.2	1	0.7	10	43.5
		HAdV-C	SAdVGroup43/45	10	7.4	0	0.0
		HAdV-E	SAdVGroup39/25/26	9	6.6	8	34.8

* = unclassified.

### Herpesviruses

Fecal samples from both chimpanzees and gorillas were positive for herpesviruses. 25.8% (41/159) were positive for at least one herpesvirus, and 12 different herpesviruses, all beta or gammaherpesviruses, were detected (Table 1).

Gorilla lymphocryptovirus, GgorLCV1, a gammaherpesvirus, was the most commonly detected herpesvirus and was found in 20.1% (32/159) of individuals. It was the only herpesvirus found in both chimpanzees (13%) and gorillas (21.3%) (Table 1). The GgorLCV1 sequence obtained from the chimpanzees was 99.4% identical to sequences obtained from gorillas. Cytomegaloviruses (CMV), a type of betaherpesvirus, were also detected but were less common (11.9%; 19/159) than GgorLCV1 (Table 1 and S1 Table). Feces from two CMV-positive gorillas, WDG61 and WDG39, each contained two different CMVs (S1 Table). Previous work has shown that within the CMVs, two chimpanzee and gorilla co-speciation clades (CG1 and CG2) exist, which contain closely related gorilla and chimpanzee viruses [18]. Nine individuals were positive for CMV in the CG1 clade. GgorCMV1.1 was identified in eight individual gorillas; phylogenetic analysis assigned these viruses into two groups within the CG1 clade (GgorCMV1.1 Group 1 (n = 4) and a separate group that we have called GgorCMV1.1 Group 2 (n = 4)) (Fig. 1). A ninth individual was positive for *Pan troglodytes* CMV (PtroCMV1.1), another cytomegalovirus in the CG1 clade. CMVs in the CG2 clade were detected in 12 individual gorillas and were assigned to four groups, GgorCMV2.2 Group 1 (n = 2) and a second group that has not previously been described that we have called GgorCMV2.2 Group 2 (n = 4), GgorCMV2.1 (n = 5), and PtroCMV2.1 (n = 1) (Fig. 1).

### Adenoviruses

**Fig 2:**
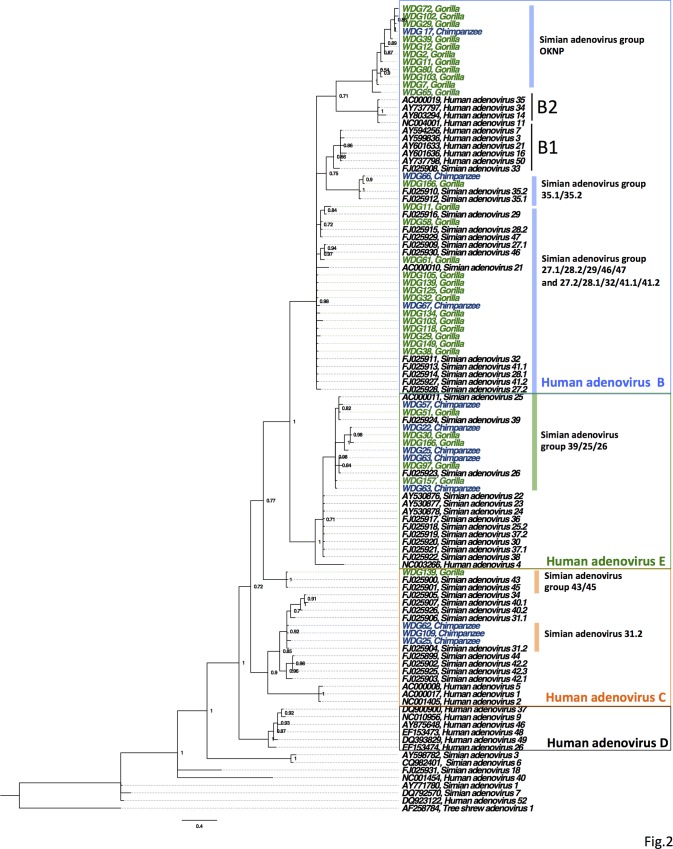
Phylogenetic tree of adenovirus lineages found in gorillas and chimpanzees. The tree was constructed through a nucleotide alignment of the adenovirus polymerase gene using Bayesian analysis. Sample identification (WDG number) and Genbank accession numbers are shown. New AdV sequences are indicated in green for gorillas, and chimpanzees in blue. The colored vertical bars indicate the names assigned to each virus group used in the analysis. The colored boxes denote adenovirus species clades. *Tree shrew AdV-1* was used as the outgroup. All SAdVGroupOKNP sequences are available in GenBank under the following accession numbers: KJ780330-KJ780358.

Adenoviruses were present in feces from 48.4% (77/159) of individuals; 69.6% (16/23) of the chimpanzees and 44.9% (61/136) of the gorillas were positive (Table 1). Gorilla and chimpanzee adenoviruses clustered within one of seven adenovirus groups in the HAdV-B, HAdV-C, or HAdV-E clades (Fig. 2). Of these, identified members in five groups were found in both chimpanzees and gorillas (Table 1). A previously unidentified group, which we named Simian Adenovirus B Group OKNP (SAdVGroupOKNP) was the most common adenovirus group and was detected in 20.1% (32/159) of tested fecal samples, and all but two positives were from gorillas (Table 1). Phylogenetically, SAdVGroupOKNP clustered with B2 human adenoviruses but shared less than 90% nucleotide identity with all other known human and simian sequences from HAdV-B. Two groups of viruses, SAdV31.2 and SAdVGroup 43/45, members of adenovirus HAdV-C, were also identified. There was a significantly greater occurrence of SAdV31.2 in chimpanzee feces (OR = 95[95% CI = 11, 810]) than in gorilla feces, but SAdVGroup 43/45 was only found in gorillas. Lastly, a group belonging to HAdV-E was also identified. This group, SAdVGroup 39/35/26 E, was also more likely to be found in chimpanzees than gorillas (OR = 6.5[95% CI = 2.1, 20]). While SAdV31.2 and SAdVGroup 39/35/26 E were found to be significantly more prevalent in chimpanzees, adenovirus B groups (SAdVGroupOKNP, SAdVGroup27.1/28.2/29/46/47, SAdVGroup27.2/28.1/32/41.1/41.2, or SAdVGroup35.1/35.2) were statistically more prevalent in gorillas (OR = 2.4[95% CI = 1.3, 14]). Adenovirus co-detection, where more than one type of adenovirus was detected, was also not statistically different among chimpanzees and gorillas, and occurred in 26.0% (6/23) of chimpanzees and 14.7% (20/136) of gorillas.

**Fig 3:**
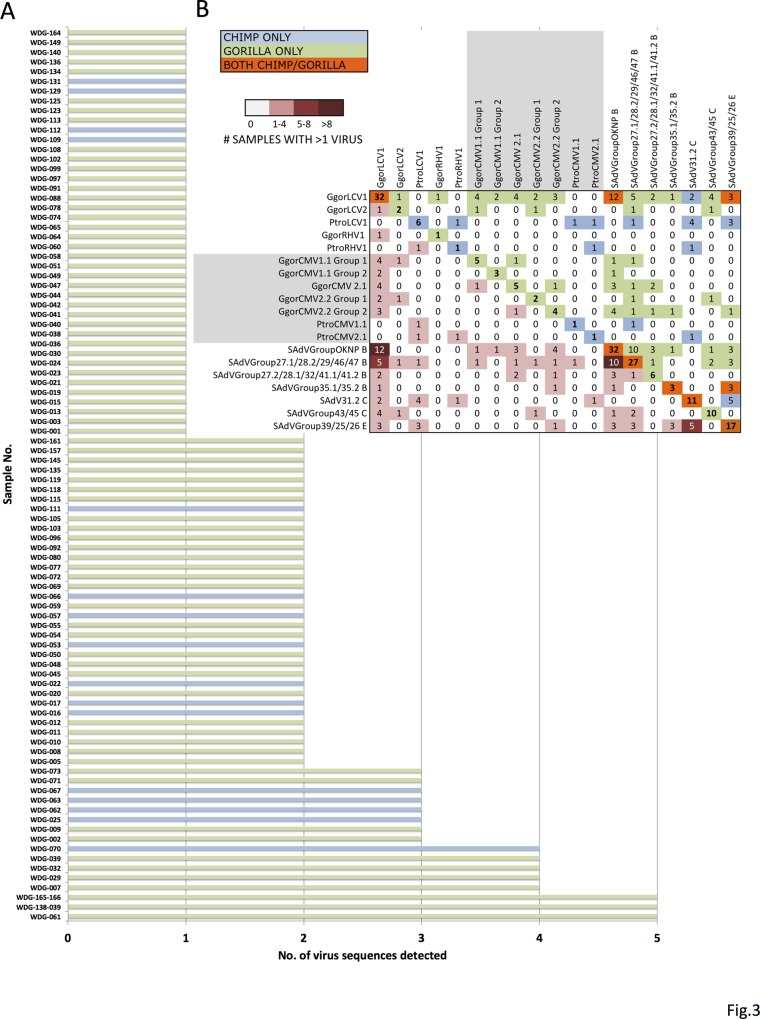
Viral co-detection in chimpanzee and gorilla fecal samples. A. Graph depicting the type and number of viruses detected in fecal samples from individual great apes. Gorillas are highlighted in green and chimpanzees are highlighted in blue. B. Matrix analysis showing all 19 detected viruses or viral groups and the number of pair-wise combinations with each other. On the left side of the matrix is a heat map showing the number of individuals positive for co-detection of two viruses as is indicated in the red color key. For example, feces from 12 individuals, highlighted in maroon (>8 samples detected with that virus combination), were positive for both GgorLCV1 and SAdVGroupOKNP. The upper right half of the matrix (mirror image of lower left) are the number of individuals that tested positive for co-detection, with colors indicating which species, or if both were affected.

Multiple viral sequences were detected in feces from 31.4% (50/159) of individuals. 27.9% (38/136) of the gorilla and 52.2% (12/23) of the chimpanzee fecal samples contained more than one virus. The distribution of positive results per individual, ranked from one to five viruses found, is shown in Fig. 3A. The data from two positive individuals that were resampled are presented in S1 Table. The remaining third individual (WDG93 and WDG95) was negative for all viruses tested. Matrix analysis comparing the presence of each of the 19 different viruses detected within individuals was performed (Fig. 3B). In individuals positive for more than one virus, GgorLCV1 and SAdVGroupOKNP was the most common virus combination detected (Fig. 3B, dark maroon cell). In the 12 individuals positive for both of these viruses, one was from a chimpanzee and 11 were from gorillas (S1 Table). SAdVGroup 27.1/28.2/29/46/47 and SAdVGroupOKNP virus combinations were also relatively common and found in 10 gorillas. Other combinations of viruses were also seen. 94.7% (18/19) of the CMV-positive individuals and 84.6% (33/39) of the LCV-positive fecal samples from individual apes contained another adenovirus or herpesvirus (S1 Table). LCV’s were found in combination with all 19 detected virus or viral groups. Overall, co-detection of HAdV-B or LCV was found in 52.6% (10/19) or 89.5% (17/19) respectively of the CMV-positive individuals.

### Estimating viral richness

**Fig 4:**
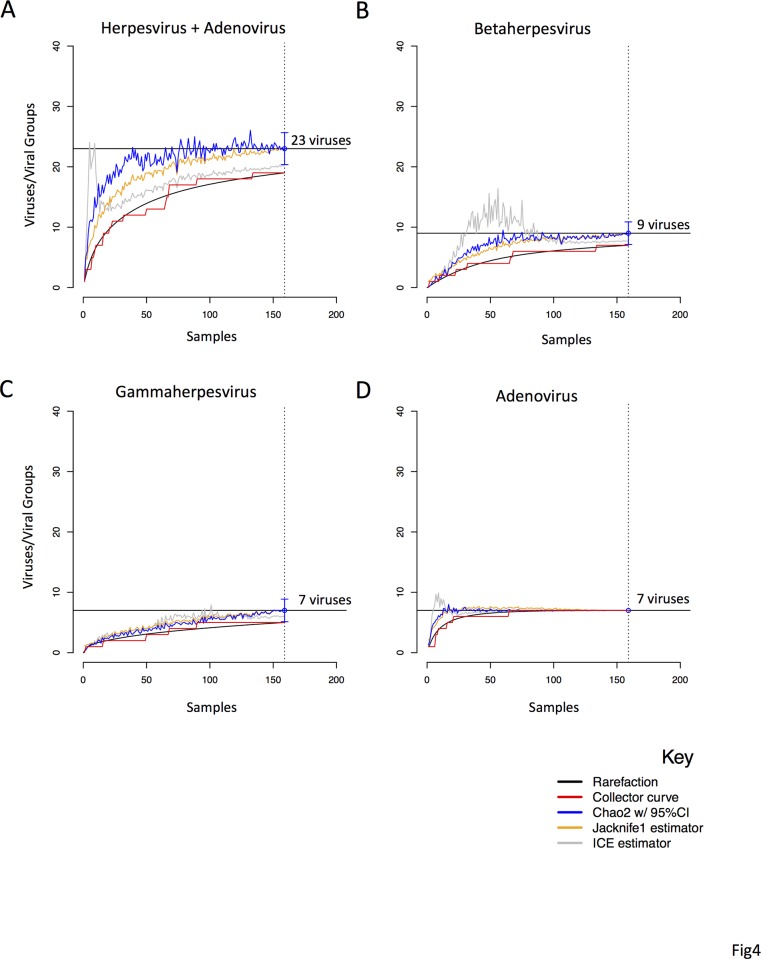
Estimating viral richness of herpesviruses and adenoviruses in great apes of the Sangha region. A. We calculated the total viral richness in chimpanzees and gorillas associated with the Adenoviridae and Herpesviridae families. ICE and Jackknife estimators are shown as orange and grey curves respectively. The refraction curve (black) shows the number of virus groups or viruses as a function of the number of samples, and the collector curve (red) shows the accumulation of viruses found with increasing sample size. The Chao2 estimator (blue) shows the number of viruses that are estimated in the ape population with 95% confidence intervals indicated by the brackets. The horizontal bar shows the number of viruses estimated in the analysis. In B, C, and D separate richness analyses were done for the Herpesviridae subfamilies (*Betaherpesvirinae* and *Gammaherpesvirinae*) and *Adenoviridae*.

In chimpanzees and gorillas, the estimated number of viruses or viral groups present in our sample population was 23 [95%CI = 20, 26] (Fig. 4). Our result shows that when estimating the viral richness for both herpesviruses and adenoviruses, the Chao 2 estimator began to plateau at 100 individuals, and was stable by 125. We estimate that we captured 83% (19/23) of the total viruses or viral groups in our study population of chimpanzees and gorillas. Looking at betaherpesviruses alone, viral richness was estimated to be nine, of which 78% (7/9) were captured in our study. For gammaherpesviruses, viral richness was estimated to be seven, of which 71% (5/7) were captured in our study. Because we could not differentiate between all the strains and had to bin our adenovirus results into closely related groups, we were unable to estimate the true viral richness for individual adenovirus strains and instead estimate the viral richness of these subgroups. For example, the viral richness of adenoviruses in this population was estimated to be seven groups, of which 100% were detected. However, this is an underestimate of the total number of actual strains present. Also, it is important to point out that we analyzed one gene for adenovirus, the polymerase gene, which is highly conserved. We did not compare the less conserved hexon gene [21]. We therefore cannot rule out the co-presence of other adenovirus serotypes belonging to the same species in our study. Additional the sequences from this study were obtained through cloning of the PCR product and sequencing multiple clones, which has the potential to underestimate viral diversity based on the number of times the PCR was repeated and the number of clones that were sequenced. We did not test the viral richness of individual ape species because our sample size for chimpanzees was small.

Because ultralow temperatures are recommended for long term storage of samples in RNA Later and our samples were stored at ambient temperatures (>4°C) for extended periods, test result data were analyzed to determine if the number of viruses retrieved was associated with sample storage duration. Using univariate linear regression on all data combined, a statistically significant negative association (p<0.05) was found where decreasing numbers of viruses were recovered as sample storage duration increased. Ambient storage duration of 1,000 days or greater reduced the mean number of detected viruses by 1.2 or more (S1 Fig.). Our results suggest that if samples are immediately tested (no ambient storage and processing immediately after collection) detection of an average of 2.0 viruses/sample would be expected. We did not detect a significant association between the total amount of nucleic acid recovered and ambient storage time (data not shown). Because the number of viruses detected per sample declined with prolonged storage time, the probability of virus detection was not uniform across all samples, and therefore our overall data may represent an underestimation of viral prevalence. However, there was no statistically significant negative correlation with viral detection and ambient temperature if we narrowed our samples to those stored between 130 and 750 days (S1 Fig.). Viral discovery curves were therefore repeated with only those samples stored for less than 750 days. In this analysis, the estimated viral richness for betaherpesvirus and adenoviruses was similar to that for all samples combined (S2 Fig.). However, the Chao2 estimator failed to stabilize for total viruses and gammaherpesvirus. Taken together, these results suggest that our estimate of viral richness of adenovirus subgroups and betaherpesvirus has not been influenced by ambient storage, however gammaherpesvirus viral richness may have been underestimated in our total estimation of viral richness of 23 viruses.

## Discussion

In order to understand natural patterns of health and disease and to monitor populations of free-ranging chimpanzees and gorillas for pathogen presence or emergence, baseline information on the natural viral diversity in these populations is needed. The goals of this study were therefore to investigate the diversity of viruses in free-ranging gorillas and chimpanzees from the Sangha region of the Republic of Congo and estimate viral richness in these populations through pathogen detection in fecal samples. We detected 19 viruses or viral groups, including a previously undescribed group of HAdV-B and 2 groups of cytomegaloviruses. Based on viral diversity curves, we estimate that we detected ∼80% of the viruses or viral groups within the Adenoviridae and Herpesviridae families that are circulating in western lowland gorillas and chimpanzees in the Sangha region near OKNP. Our estimation of herpesvirus and adenovirus richness in these two ape populations, although limited to a single region, provides important information about the types of viruses that circulate in these species and this population. This estimate does not apply to the viral richness that could be determined in ape populations elsewhere in the world or in captivity, but our results can provide opportunities for future comparison. Our study also does not address novel viruses that can only be detected using unbiased high throughput sequencing methods.

Adenoviruses are double-stranded DNA viruses that can infect a wide range of vertebrate hosts and cause a variety of diseases including mild to life-threatening respiratory and gastrointestinal disease. Human and non-human primates shed adenoviruses in their stool [21,36–38]. In one study, adenovirus DNA was identified in 40% of stool samples from wild chimpanzees in Cameroon, and the Democratic Republic of Congo (DRC), and captive gorillas maintained in zoos with a much higher prevalence of shedding (36–100%) in the latter [21]. Herein we report prevalence statistics, but because of potential deterioration issues with the samples these are assumed to be an underestimate of the true value in our sampled population. In our study region in the Republic of Congo, adenovirus shedding was also common in free-ranging great apes, being present in fecal samples from 68% of chimpanzees and 45% of gorillas. Results in our chimpanzee study population were higher than in the previous Cameroon/DRC study. This could be due to differences in sample storage conditions, population density, seasonality, or geographic region, but is less likely due to laboratory differences, since both studies used the same PCR assay [21]. Of the three adenovirus species detected in the current study (HAdV-B, HAdV-C, and HAdV-E, all were found in both chimpanzees and gorillas. Previous studies have only detected HAdV-E in chimpanzees, bonobos and humans [21,37]; thus, our findings of HAdV-E in both gorillas and chimpanzees support the possibility for an earlier cross-species transmission event in this population. Adenoviruses are thought to co-evolve with their host species and usually do not cross the species barrier [39–41]. However, genetic and serological studies support the concept of cross-species adenovirus transmission between non-human primates and humans [37,42,43]. Additional surveys are warranted to better understand the likelihood of cross-species transmission events in primates and determine if recombination and generation of hybrid adenoviruses, which have developed in captive settings [21], could occur in the wild. This type of information would promote a better understanding of the risk for zooanthroponotic transmission, and whether adenoviruses in wild gorillas and chimpanzees are a risk factor for disease emergence in humans in situations where humans are working closely with captive primates or living in communities that border free-ranging gorilla or chimpanzee habitat.

Detection of fragments from multiple different adenoviruses in gorilla and chimpanzee feces was a significant finding in our study. Feces from 16.4% (26/159) of individuals, six chimpanzees and 20 gorillas, contained more than one type of adenovirus from seven different groups of three different species. This adds significantly to our understanding of the different adenoviruses to which gorilla and chimpanzees are exposed in or east of OKNP. Adenovirus coinfection associated with illness has been well documented in humans. Human adenovirus account for about 8% of clinically relevant viral disease globally, are divided into 52 serotypes from seven species [44,45]. About 50% of the 52 identified human adenovirus serotypes are known to cause illness [46]. Adenovirus infections, such as HAdV-14 (B2) cause respiratory disease and pneumonia [47]. Other adenoviral disease, including conjunctivitis, hepatitis and diarrhea [48], are described in children, immunocompromised people, and in patients with multiple co-infections [46]. Two recent studies showed a high rate of adenoviral co-infection with HAdV-B and E in humans with acute respiratory disease [44,49]. In the great apes described herein, adenoviral co-detections included combinations of HAdV-B/C and HAdV-B/E. In contrast to humans, very little is known about the pathogenicity of adenoviruses or the significance of their co-infections in great apes and other non-human primates [21,38,43,50]. Given current limits in our understanding of the ecology, including cross species transmission, our results underscore the importance of developing a more comprehensive understanding of adenoviral infections in great apes.

Herpesviruses are divided into three distinct subfamilies known as *Alphaherpesvirinae*, *Betaherpesvirinae*, and *Gammaherpesvirinae*, based on biological and molecular properties and genomic sequence divergence [51]. Cytomegaloviruses (CMVs) and lymphocryptoviruses (LCVs) are members of the Beta- and Gammaherpesvirinae subfamilies respectively, that infect mammals, human and non-human primates. Although host-virus co-divergence is thought to be the primary mode of herpesvirus evolution, cross-species transmission events have been known to occur. For example, humans infected with macaque simplex virus (Herpes B virus) can develop severe illness or death [52], and simian CMV has been shown to infect human cells [53,54].

Because of the close evolutionary relationship between great apes and humans, herpesviruses of apes are of particular interest for public health and those that impact great apes directly are important for species conservation. Several new CMVs have recently been characterized in both chimpanzees and gorillas [18]. These discoveries have led to the identification of two new co-speciation clades of CMV (CG1 and CG2) that are thought to have evolved through a horizontal transmission event between chimpanzees and gorillas 6–8 million years ago [18]. DNA fragments from seven CMVs were identified in the non-human great ape feces from this study. These included two previously undescribed CMVs that we designated GgorCMV1.1 Group 2 and GgorCMV2.2 Group 2, within the CG1 and CG2 clades, respectively. All of the CMV viruses detected in our study were found in feces from either gorillas or chimpanzees (no detection of the same viral fragment in both species). GgorLCV1, a gammaherpesvirus, was the only herpesvirus found in both gorillas and chimpanzees.

Epstein-Barr virus (EBV, human lymphocryptovirus) and CMV are the most common cause of infectious mononucleosis (IM) and illnesses resembling IM in children, and can lead to severe fatigue, fever, pharyngitis, lymphadenopathy and recurrent microbial infection [55,56]. Co-infections of these two herpesvirus in the context of other viral infections, such as an additional herpes or adenovirus, can worsen symptoms and illness [55, 57], and reactivation of CMV has been suggested to be a factor leading to exacerbation of disease induced by other viral agents [18]. In the current study, 95% of the CMV-positive and 85% of the LCV-positive ape fecal samples contained more than one virus, either another herpesvirus and/or adenovirus, which suggests that CMV and LCV are primarily shedding into the feces in the context of these other viruses. Because CMV/LCV/adenovirus co-association is so tightly correlated in our study, pre-screening of ape feces for these viruses could be used to identify animals or groups of animals with co-infections. Evaluating these animals for disease outcomes may be a useful strategy to determine if viral co-infection reflects similar outcomes as seen in humans.

Understanding the diversity of pathogens to which endangered gorillas and chimpanzees are exposed is important in defining health risks that impact population dynamics and conservation efforts. Our results provide initial information about the presence and diversity of adenoviruses and herpesviruses in and near OKNP to which wild gorillas and chimpanzees, as well as humans or other species that live in the region, are exposed. Limitations of the current study are a likely underestimation of viral diversity in the sample ape population due to fecal degradation of samples stored for long term that resulted in reduced DNA and RNA viral detection. Subsequent studies across this and other regions in Africa, that incorporate unbiased sequencing technologies, will be valuable for estimating the true viral diversity in great ape species as a whole, not just at the population level. In addition, continuous monitoring of these endangered species is necessary to understand the expression and outcomes of infection, co-infection, and cross-species transmission; all crucial steps for defining health-related conservation threats and guiding conservation management actions.

## Supporting Information

S1 Dataset FASTA file. DNA sequence alignment of herpesvirus glycoprotein B sequences used for phylogenetic analysis in Fig. 1.
(FASTA)

S2 Dataset FASTA file. DNA sequence alignment of adenovirus DNA polymerase sequences used for phylogenetic analysis in Fig. 2.
(FASTA)

S1 Fig Association of viral detection and days stored at ambient temperature. A. Univariate linear regression showing a statistically significant negative association (p<0.05) with the number of viruses recovered and the number of days the samples were stored at ambient temperature. Ambient storage duration of 1,000 days or greater reduced the mean number of viruses detected by 1.2 or more. B. Univariate linear regression showing no statistically significant negative association with the virus count and the number of days the samples were stored at ambient temperature when analyzing samples stored up to 750 days.
(TIFF)

S2 Fig Viral richness in samples stored at ambient temperature for less time. Estimated viral richness curves for chimpanzees and gorillas associated with the Adenoviridae and Herpesviridae families repeated as in Fig. 4, but with only samples stored for less than 750 days.
(TIFF)

S1 Table Summary of positive results. Positive PCR results obtained for each individual grouped by Gammaherpesvirinae, Betaherpevirinae and Adenoviridae subfamilies or family, the total number of viruses detected for each individual, and type of animal each sample was derived from.
(DOCX)

S2 Table BLASTn results of viral alignments with those found in GenBank. National Center for Biotechnology Information: http://www.blast.ncbi.nlm.nih.gov/Blast.cgi.
(DOCX)

## References

[1] Bermejo M, Rodríguez-Teijeiro J, Illera G, Barroso A, Vilà C, Walsh P. Ebola outbreak killed 5000 gorillas. Science. 2006; 314: 1564 17158318 10.1126/science.1133105

[2] Jones K, Patel N, Levy M, Storeygard A, Balk D, Gittleman J, et al Global trends in emerging infectious diseases. Nature. 2008; 451: 990–993. doi: 10.1038/nature06536 18288193 10.1038/nature06536 PMC5960580

[3] Karesh WB, Dobson A, Lloyd-Smith JO, Lubroth J, Dixon MA, Bennett M, et al Ecology of zoonoses: natural and unnatural histories. The Lancet. 2012; 380: 1136–1145. 10.1016/S0140-6736(12)61678-X PMC7138068 23200502

[4] Liu W, Li Y, Learn G, Rudicell R, Robertson J, Keele B, et al Origin of the human malaria parasite *Plasmodium falciparum* in gorillas. Nature. 2010; 467: 420–425. doi: 10.1038/nature09442 20864995 10.1038/nature09442 PMC2997044

[5] Prugnolle F, Durand P, Neel C, Ollomo B, Ayala F, Arnathau C, et al African great apes are natural hosts of multiple related malaria species, including *Plasmodium falciparum* . Proc Natl Acad Sci USA. 2010; 107: 1458–1463. doi: 10.1073/pnas.0914440107 20133889 10.1073/pnas.0914440107 PMC2824423

[6] Olson S, Reed P, Cameron K, Ssebide B, Johnson C, Morse S, et al Dead or alive: animal sampling during Ebola hemorrhagic fever outbreaks in humans. Emerg Health Threats J. 2012 5: 3402/ehtj.v3405i3400.9134. 10.3402/ehtj.v5i0.9134 PMC3342678 22558004

[7] Reed P, Mulangu S, Cameron K, Ondzie A, Joly D, Bermejo M, et al A new approach for monitoring ebolavirus in wild great apes. PLOS Negl Trop Dis. 2014; 8: e3143 doi: 10.1371/journal.pntd.0003143 25232832 10.1371/journal.pntd.0003143 PMC4169258

[8] Wolfe ND, Dunavan CP, Diamond J. Origins of major human infectious diseases. Nature. 2007; 447: 279–283. 17507975 10.1038/nature05775 PMC7095142

[9] Kaur T, Singh J, Tong S, Humphrey C, Clevenger D, Tan W, et al Descriptive epidemiology of fatal respiratory outbreaks and detection of a human-related metapneumovirus in wild chimpanzees (*Pan troglodytes*) at Mahale Mountains National Park, Western Tanzania. Am J Primatol. 2008; 70: 755–765. doi: 10.1002/ajp.20565 18548512 10.1002/ajp.20565 PMC7159556

[10] Palacios G, Lowenstine LJ, Cranfield MR, Gilardi KV, Spelman L, Lukasik-Braum M, et al Human metapneumovirus infection in wild mountain gorillas, Rwanda. Emerg Infect Dis. 2011; 17: 711–713. doi: 10.3201/eid1704.100883 21470468 10.3201/eid1704.100883 PMC3377396

[11] Ryan S and Walsh P. Consequences of non-intervention for infectious disease in African great apes. PLOS One. 2011; 6: e29030 doi: 29010.21371/journal.pone.0029030 22216162 10.1371/journal.pone.0029030 PMC3245243

[12] Cooper N and Nunn CL. Identifying future zoonotic disease threats: Where are the gaps in our understanding of primate infectious diseases? Evolution, Medicine, and Public Health. 2013: 27–36. 10.1093/emph/eot001 PMC3868449 24481184

[13] Oates JF, Tutin CEG, Humle T, Wilson ML, Baillie J, Balmforth Z, et al. *Pan troglodytes*. In: IUCN 2013. IUCN Red List of Threatened Species.

[14] Walsh PD, Tutin CEG, Oates JF, Baillie J, Maisels F, Stokes E, et al. *Gorilla gorilla*. In: IUCN 2013. IUCN Red List of Threatened Species.

[15] Devos C, Sanz C, Morgan D, Onononga J, Laporte N, Huynen MC. Comparing ape densities and habitats in northern Congo: surveys of sympatric gorillas and chimpanzees in the Odzala and Ndoki regions. Am J Primatol. 2008; May: 439–451. 10.1002/ajp.20514 18176937

[16] Lilly AA, Mehlman PT, Doran D. Intestinal parasites in gorillas, chimpanzees, and humans at Mondika Research Site, Dzanga-Ndoki National Park, Central African Republic. International Journal of Evolutionary Biology. 2002; 23: 555–573.

[17] Chmielewicz B, Goltz M, Ehlers B. Detection and multigenic characterization of a novel gammaherpesvirus in goats. Virus Res. 2001; 75: 87–94. 11311431 10.1016/s0168-1702(00)00252-5

[18] Leendertz F, Deckers M, Schempp W, Lankester F, Boesch C, Mugisha L. et al Novel cytomegaloviruses in free-ranging and captive great apes: phylogenetic evidence for bidirectional horizontal transmission. J Gen Virol. 2009; 90: 2386–2394. doi: 10.1099/vir.0.011866-0 19553394 10.1099/vir.0.011866-0

[19] VanDevanter D, Warrener P, Bennett L, Schultz E, Coulter S, Garber RL, et al Detection and analysis of diverse herpesviral species by consensus primer PCR. J Clin Microbiol. 1996; 34: 1666–1671. 8784566 10.1128/jcm.34.7.1666-1671.1996 PMC229091

[20] Prepens S, Kreuzer K, Leendertz F, Nitsche A, Ehlers B. Discovery of herpesviruses in multi-infected primates using locked nucleic acids (LNA) and a bigenic PCR approach. Virology Journal. 2007; 4: 1–14. 17822523 10.1186/1743-422X-4-84 PMC2014757

[21] Roy S, Vandenberghe L, Kryazhimskiy S, Grant R, Calcedo R, Yuan X, et al Isolation and characterization of adenoviruses persistently shed from the gastrointestinal tract of non-human primates. PLOS Pathog. 2009; 5: e1000503 doi: 10.1371/journal.ppat.1000503 19578438 10.1371/journal.ppat.1000503 PMC2698151

[22] Zhai J, Palacios G, Towner J, Jabado O, Kapoor V, Venter M, et al Rapid molecular strategy for filovirus detection and characterization. J Clin Microbiol. 2007; 45: 224–226. 17079496 10.1128/JCM.01893-06 PMC1828965

[23] Tong S, Chern S, Li Y, Pallansch M, Anderson L. Sensitive and broadly reactive reverse transcription-PCR assays to detect novel paramyxoviruses. J Clin Microbiol. 2008; 46: 2652–2658. doi: 10.1128/JCM.00192-08 18579717 10.1128/JCM.00192-08 PMC2519498

[24] Quan P, Firth C, Street C, Henriquez J, Petrosov A, Tashmukhamedova A, et al Identification of a severe acute respiratory syndrome coronavirus-like virus in a leaf-nosed bat in Nigeria. mBio. 2010; 1: e00208–00210. doi: 10.1128/mBio.00208-10 21063474 10.1128/mBio.00208-10 PMC2975989

[25] Watanabe S, Masangkay J, Nagata N, Morikawa S, Mizutani T, Fukushi S, et al Bat coronaviruses and experimental infection of bats, the Philippines. Emerg Infect Dis. 2010; 16: 1217–1223. doi: 10.3201/eid1608.100208 20678314 10.3201/eid1608.100208 PMC3298303

[26] Moureau G, Temmam S, Gonzalez J, Charrel R, Grard G, de Lamballerie X. A real-time RT-PCR method for the universal detection and identification of flaviviruses. Vector Borne Zoonotic Dis. 2007; 4: 467–477. 18020965 10.1089/vbz.2007.0206

[27] Bracht A, Brudek R, Ewing R, Manire C, Burek K, Rosa C, et al Genetic identification of novel poxviruses of cetaceans and pinnipeds. Arch Virol. 2006; 3: 423–438. 16328132 10.1007/s00705-005-0679-6

[28] Kapoor A, Mehta N, Esper F, Poljsak-Prijatelj M, Quan P, Qaisar N, et al Identification and characterization of a new bocavirus species in gorillas. PLOS One. 2010; 5: e11948 doi: 10.1371/journal.pone.0011948 20668709 10.1371/journal.pone.0011948 PMC2909267

[29] Clewley J, Lewis J, Brown D, Gadsby E. A novel simian immunodeficiency virus (SIVdrl) pol sequence from the drill monkey, *Mandrillus leucophaeus* . J Virol. 1998; 72: 10305–10309. 9811781 10.1128/jvi.72.12.10305-10309.1998 PMC110619

[30] Takehisa J, Kraus M, Ayouba A, Bailes E, Van Heuverswyn F, Decker J, et al Origin and biology of simian immunodeficiency virus in wild-living western gorillas. J Virol. 2009; 83: 1635–1648. doi: 10.1128/JVI.02311-08 19073717 10.1128/JVI.02311-08 PMC2643789

[31] Wroblewski EE, Murray CM, Keele BF, Schumacher-Stankey JC, Hahn BH, Pusey AE. Male dominance rank and reproductive success in chimpanzees, *Pan troglodytes schweinfurthii* . Animal Behavior. 2009; 77: 873–885. 19498952 10.1016/j.anbehav.2008.12.014 PMC2689943

[32] Kocher TD, Thomas WK, Meyer A, Edwards SV, Pääbo S, Villablanca FX, et al Dynamics of mitochondrial DNA evolution in animals: Amplification and sequencing with conserved primers. Proc Natl Acad Sci USA.1989; 86: 6196–6200. 2762322 10.1073/pnas.86.16.6196 PMC297804

[33] Huelsenbeck JP, Ronquist F. MRBAYES: Bayesian inference of phylogeny. Bioinformatics. 2001;17: 754–755. 11524383 10.1093/bioinformatics/17.8.754

[34] R Development Core Team (2011) R: A language and environment for statistical computing. R Foundation for Statistical Computing. Available: http://www.R-project.org/ Vienna, Austria.

[35] Anthony SJ, Epstein JH, Murray KA, Navarrete-Macias I, Zambrana-Torrelio CM, Solovyov A, et al A strategy to estimate unknown viral diversity in mammals. mBio. 2013; 4: e00598–00513. doi: 10.1128/mBio.00598-13 24003179 10.1128/mBio.00598-13 PMC3760253

[36] Noble RT, Allen SM, Blackwood AD, Chu W, Jiang SC, Lovelace GL, et al Use of viral pathogens and indicators to differentiate between human and non-human fecal contamination in a microbial source tracking comparison study. J Water Health Dec. 2003; 1: 195–207. 15382724

[37] Wevers D, Metzger S, Babweteera F, Bieberbach M, Boesch C, Cameron K, et al Novel Adenoviruses in Wild Primates: A High Level of Genetic Diversity and Evidence of Zoonotic Transmissions. J Virol. 2011; 85: 10774 doi: 10.1128/JVI.00810-11 21835802 10.1128/JVI.00810-11 PMC3187507

[38] Wevers D, Leendertz F, Scuda N, Boesch C, Robbins M, Head J, et al A novel adenovirus of Western lowland gorillas (*Gorilla gorilla gorilla*). Virol J. 2010; 7: doi: 10.1186/1743-1422X-1187-1303 10.1186/1743-422X-7-303 PMC2989969 21054831

[39] Benkö M and Harrach B. Molecular evolution of adenoviruses. Curr Top Microbiol Immunol. 2003; 272: 3–35. 12747545 10.1007/978-3-662-05597-7_1

[40] Davison A, Benkö M, Harrach B. Genetic content and evolution of adenoviruses. J Gen Virol. 2003 84: 2895–2908. 14573794 10.1099/vir.0.19497-0

[41] Benkö M, Harrach B, Kremer E. Do nonhuman primate or bat adenoviruses pose a risk for human health? Future Microbiol. 2014; 9: 269–272. doi: 10.2217/fmb.13.170 24762301 10.2217/fmb.13.170

[42] Chen E, Yagi S, Kelly K, Mendoza S, Maninger N, Rosenthal A, et al Cross-species transmission of a novel adenovirus associated with a fulminant pneumonia outbreak in a New World monkey colony. PLOS Pathog. 2011; 7: e1002155 doi: 10.1371/journal.ppat.1002155 21779173 10.1371/journal.ppat.1002155 PMC3136464

[43] Chiu C, Yagi S, Lu X, Yu G, Chen E, Liu M, et al A novel adenovirus species associated with an acute respiratory outbreak in a baboon colony and evidence of coincident human infection. mBio. 2013; 4: e00084–00013. doi: 10.1128/mBio.00084-13 23592261 10.1128/mBio.00084-13 PMC3634605

[44] Vora GJ, Lin B, Gratwick K, Meador C, Hansen C, Tibbetts C, et al Co-infections of adenovirus species in previously vaccinated patients. Emerg Infect Dis. 2006; 12:921–930. 16707047 10.3201/eid1206.050245 PMC3373024

[45] Virus taxonomy: classification and nomenclature of viruses: Ninth Report of theInternational Committee on Taxonomy of Viruses Ed: King A.M.Q., Adams M.J., Carstens E.B. and Lefkowitz E.J. San Diego: Elsevier *Adenoviridae* Chapter. 2012 pp. 125–141.

[46] Gray G, McCarthy T, Lebeck M, Schnurr D, Russell K, Kajon AE et al Genotype prevalence and risk factors for severe clinical adenovirus infection, United States 2004–2006. Clin Infect Dis. 2007;45: 1120–1131. 17918073 10.1086/522188 PMC2064001

[47] Lewis P, Schmidt M, Lu X, Erdman D, Campbell M, Thomas A, et al A community-based outbreak of severe respiratory illness caused by human adenovirus serotype 14. J Infect Dis. 2009; 199: 1427–1434. doi: 10.1086/598521 19351259 10.1086/598521

[48] Ruuskanen O, Meurman O, Akusjarvi G. Adenoviruses In: Richman DD WR, Hayden FG, eds., editor. Clinical Virology. New York: Churchill Livingstone.1997 pp. 1355.

[49] Wang SL, Chi CY, Kuo PH, Tsai HP, Wang SM, Liu CC, et al High-incidence of human adenoviral co-infections in taiwan. PLOS One. 2013; 8: e75208 doi: 75210.71371/journal.pone.0075208 24073254 10.1371/journal.pone.0075208 PMC3779158

[50] Duncan M, Cranfield M, Torano H, Kuete H, Lee G, Glenn A, et al Adenoviruses isolated from wild gorillas are closely related to human species C viruses. Virology. 2013; 444: 119–123. doi: 110.1016/j.virol.2013.1005.1041 23806387 10.1016/j.virol.2013.05.041

[51] Davison A. Evolution of the herpesviruses. Vet Microbiol. 2002; 86: 69–88. 11888691 10.1016/s0378-1135(01)00492-8

[52] Wertheim J, Smith M, Smith D, Scheffler K, Kosakovsky Pond S. Evolutionary origins of human herpes simplex viruses 1 and 2. Mol Biol Evol. 2014; 31: 2356–2364. doi: 10.1093/molbev/msu185 24916030 10.1093/molbev/msu185 PMC4137711

[53] Lilja A, Shenk T. Efficient replication of rhesus cytomegalovirus variants in multiple rhesus and human cell types. Proc Natl Acad Sci USA. 2008; 105: 19950–19955. doi: 10.1073/pnas.0811063106 19064925 10.1073/pnas.0811063106 PMC2604975

[54] Michaels M, Alcendor D, St George K, Rinaldo C, Ehrlich G, Becich MJ, et al Distinguishing baboon cytomegalovirus from human cytomegalovirus: importance for xenotransplantation. J Infect Dis. 1997; 176: 1476–1483. 9395357 10.1086/514144

[55] Müller R, Ditzenb A, Hillea K, Stichlinga M, Ehrichtc R, Illmer T, et al Detection of herpesvirus and adenovirus co-infections with diagnostic DNA-microarrays. Journal of Virological Methods. 2009; 155: 161–166. doi: 10.1016/j.jviromet.2008.10.014 19022297 10.1016/j.jviromet.2008.10.014

[56] Wang X, Yang K, Wei C, Huang Y, Zhao D. Coinfection with EBV/CMV and other respiratory agents in children with suspected infectious mononucleosis. Virol J. 2010; 7: doi: 10.1186/1743-1422X-1187-1247 10.1186/1743-422X-7-247 PMC2949848 20858235

[57] Coaquette A, Bourgeois A, Dirand C, Varin A, Chen W, Herbein G. Mixed cytomegalovirus glycoprotein B genotypes in immunocompromised patients. Clin Infect Dis. 2004; 39: 155–161. 15307021 10.1086/421496

