# Correction: Adenovirus and Herpesvirus Diversity in Free-Ranging Great Apes in the Sangha Region of the Republic of Congo

**DOI:** 10.1371/journal.pone.0142766

**Published:** 2015-11-10

**Authors:** Tracie A. Seimon, Sarah H. Olson, Kerry Jo Lee, Gail Rosen, Alain Ondzie, Kenneth Cameron, Patricia Reed, Simon J. Anthony, Damien O. Joly, William B. Karesh, Denise McAloose, W. Ian Lipkin

William B. Karesh is not included in the author byline. He should be listed as the tenth author and affiliated with Wildlife Health and Health Policy Program, Wildlife Conservation Society, Bronx, New York, United States of America. The contributions of this author are as follows: Collected and provided fecal samples analyzed in the manuscript.
