# Correction: Adenovirus and Herpesvirus Diversity in Free-Ranging Great Apes in the Sangha Region of the Republic of Congo

**DOI:** 10.1371/journal.pone.0221845

**Published:** 2019-08-26

**Authors:** Tracie A. Seimon, Sarah H. Olson, Kerry Jo Lee, Gail Rosen, Alain Ondzie, Kenneth Cameron, Patricia Reed, Simon J. Anthony, Damien O. Joly, William B. Karesh, Denise McAloose, W. Ian Lipkin

The following information is missing from the Data Availability statement: Raw data supporting Fig 4 and S1 Fig are found in the S1 File.

S1 File is omitted from the list of Supporting Information. It can be viewed below.

## Supporting information

S1 File Raw data supporting Fig 4 and S1 Fig. (XLSX)
