# Supplementary figures and images for: Adenovirus and Herpesvirus Diversity in Free-Ranging Great Apes in the Sangha Region of the Republic of Congo
Source: PLoS One. 2015 Mar 17;10(3):e0118543. doi: 10.1371/journal.pone.0118543 (PMC4362762; doi:10.1371/journal.pone.0118543)

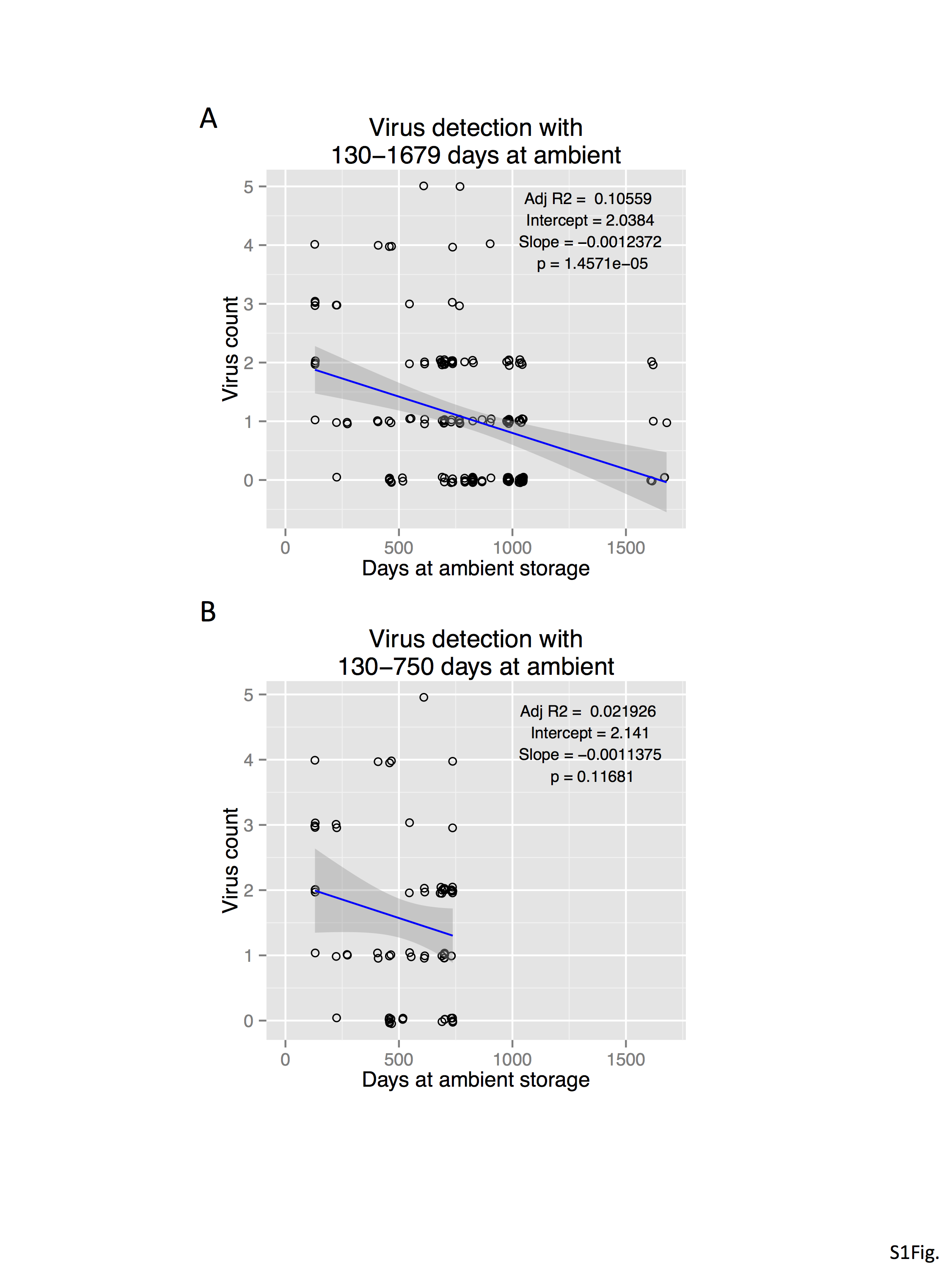

Supplement: S1 Fig — A. Univariate linear regression showing a statistically significant negative association (p<0.05) with the number of viruses recovered and the number of days the samples were stored at ambient temperature. Ambient storage duration of 1,000 days or greater reduced the mean number of viruses detected by 1.2 or more. B. Univariate linear regression showing no statistically significant negative association with the virus count and the number of days the samples were stored at ambient temperature when analyzing samples stored up to 750 days. (TIFF) [file pone.0118543.s003.tiff]

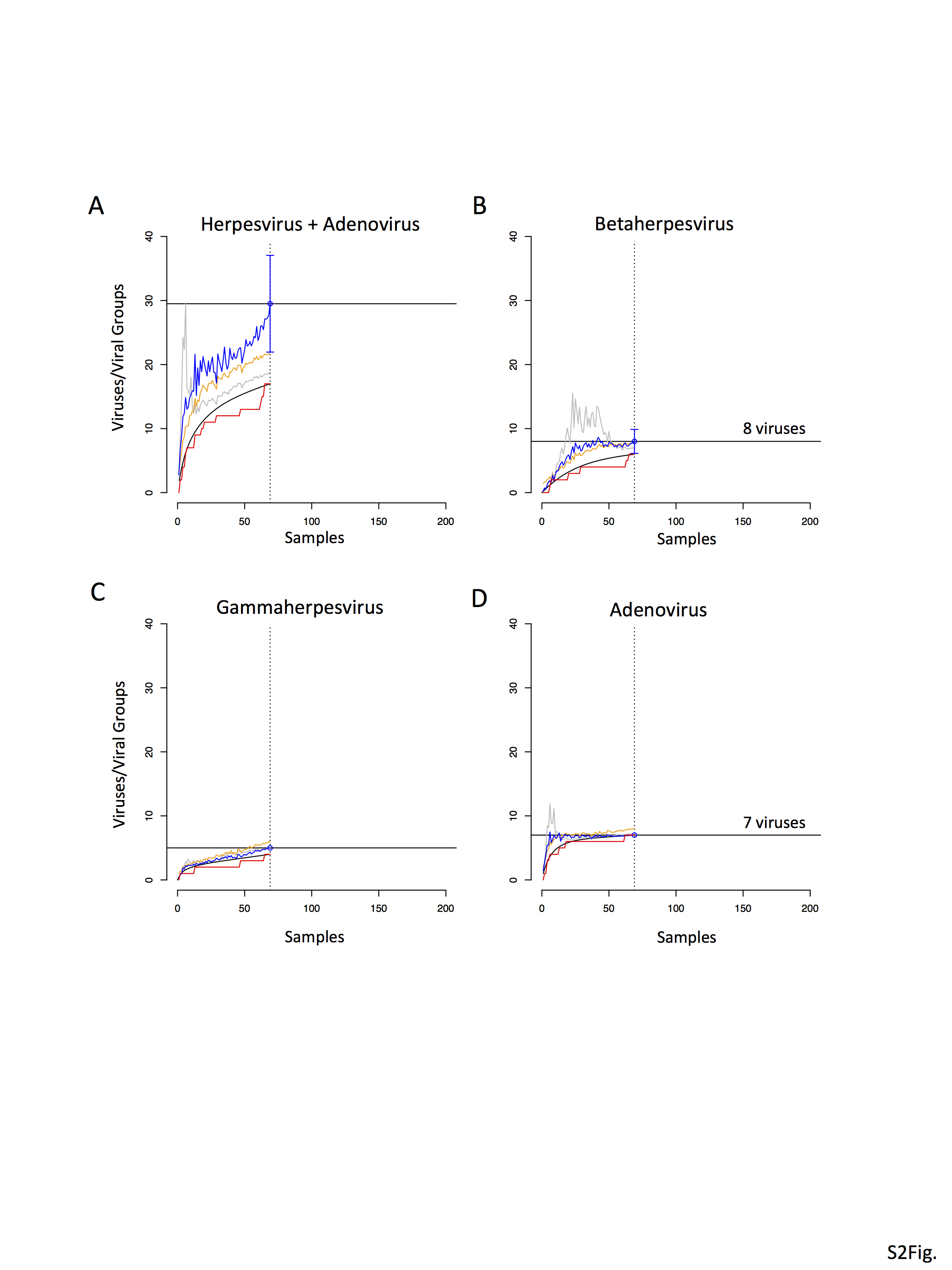

Supplement: S2 Fig — Estimated viral richness curves for chimpanzees and gorillas associated with the Adenoviridae and Herpesviridae families repeated as in Fig. 4, but with only samples stored for less than 750 days. (TIFF) [file pone.0118543.s004.tiff]
