# Supplementary material for: Adenovirus and Herpesvirus Diversity in Free-Ranging Great Apes in the Sangha Region of the Republic of Congo
Source: PLoS One. 2015 Mar 17;10(3):e0118543. doi: 10.1371/journal.pone.0118543 (PMC4362762; doi:10.1371/journal.pone.0118543)
Supplement: S1 Table — Positive PCR results obtained for each individual grouped by Gammaherpesvirinae, Betaherpevirinae and Adenoviridae subfamilies or family, the total number of viruses detected for each individual, and type of animal each sample was derived from. (DOCX) [file pone.0118543.s005.docx]

**S1 Table**

|  | GgorLCV1 | GgorLCV2 | PtroLCV1 | GgorRHV1 | PtroRHV | GgorCMV1.1 Group 1 | GgorCMV1.1 Group 2 | GgorCMV 2.1 | GgorCMV2.2 Group 1 | GgorCMV2.2 Group 2 | PtroCMV1.1 | PtroCMV2.1 | SAdVGroupOKNP | SAdVGroup27.1/28.2/29/46/47 | SAdVGroup27.2/28.1/32/41.1/41.2 | SAdVGroup35.1/35.2 | SAdV31.2 | SAdVGroup43/45 | SAdVGroup39/25/26 | **No of viruses/sample** | Chimpanzee ( c ) | Gorilla ( g ) |
| --- | --- | --- | --- | --- | --- | --- | --- | --- | --- | --- | --- | --- | --- | --- | --- | --- | --- | --- | --- | --- | --- | --- |
| WDG-061 | 1 |  |  |  |  | 1 |  | 1 |  |  |  |  | 1 | 1 |  |  |  |  |  | 5 |  | g |
| WDG-138-139 | 1 | 1 |  |  |  |  |  |  | 1 |  |  |  |  | 1 |  |  |  | 1 |  | 5 |  | g |
| WDG-165-166 | 1 |  |  |  |  |  |  |  |  | 1 |  |  | 1 |  |  | 1 |  |  | 1 | 5 |  | g |
| WDG-007 | 1 |  |  |  |  |  |  |  |  |  |  |  | 1 | 1 |  |  |  |  | 1 | 4 |  | g |
| WDG-029 | 1 |  |  |  |  |  |  |  |  |  |  |  | 1 | 1 | 1 |  |  |  |  | 4 |  | g |
| WDG-032 | 1 |  |  |  |  |  |  |  |  | 1 |  |  | 1 | 1 |  |  |  |  |  | 4 |  | g |
| WDG-039 |  |  |  |  |  |  |  | 1 |  | 1 |  |  | 1 |  | 1 |  |  |  |  | 4 |  | g |
| WDG-070 |  |  | 1 |  | 1 |  |  |  |  |  |  | 1 |  |  |  |  | 1 |  |  | 4 | c |  |
| WDG-002 | 1 |  |  |  |  |  |  | 1 |  |  |  |  | 1 |  |  |  |  |  |  | 3 |  | g |
| WDG-009 | 1 |  |  |  |  |  |  | 1 |  |  |  |  |  |  | 1 |  |  |  |  | 3 |  | g |
| WDG-025 |  |  | 1 |  |  |  |  |  |  |  |  |  |  |  |  |  | 1 |  | 1 | 3 | c |  |
| WDG-062 | 1 |  |  |  |  |  |  |  |  |  |  |  |  |  |  |  | 1 |  | 1 | 3 | c |  |
| WDG-063 |  |  | 1 |  |  |  |  |  |  |  |  |  |  |  |  |  | 1 |  | 1 | 3 | c |  |
| WDG-067 |  |  | 1 |  |  |  |  |  |  |  | 1 |  |  | 1 |  |  |  |  |  | 3 | c |  |
| WDG-071 | 1 |  |  |  |  |  | 1 |  |  |  |  |  | 1 |  |  |  |  |  |  | 3 |  | g |
| WDG-073 | 1 |  |  |  |  |  |  |  |  | 1 |  |  | 1 |  |  |  |  |  |  | 3 |  | g |
| WDG-005 | 1 |  |  |  |  |  |  |  |  |  |  |  | 1 |  |  |  |  |  |  | 2 |  | g |
| WDG-008 |  |  |  |  |  |  |  |  |  |  |  |  | 1 | 1 |  |  |  |  |  | 2 |  | g |
| WDG-010 | 1 |  |  |  |  | 1 |  |  |  |  |  |  |  |  |  |  |  |  |  | 2 |  | g |
| WDG-011 |  |  |  |  |  |  |  |  |  |  |  |  | 1 | 1 |  |  |  |  |  | 2 |  | g |
| WDG-012 |  |  |  |  |  |  |  |  |  |  |  |  | 1 |  |  |  |  |  | 1 | 2 |  | g |
| WDG-016 |  |  | 1 |  |  |  |  |  |  |  |  |  |  |  |  |  | 1 |  |  | 2 | c |  |
| WDG-017 | 1 |  |  |  |  |  |  |  |  |  |  |  | 1 |  |  |  |  |  |  | 2 | c |  |
| WDG-020 | 1 |  |  |  |  | 1 |  |  |  |  |  |  |  |  |  |  |  |  |  | 2 |  | g |
| WDG-022 |  |  | 1 |  |  |  |  |  |  |  |  |  |  |  |  |  |  |  | 1 | 2 | c |  |
| WDG-045 | 1 |  |  |  |  |  |  |  |  |  |  |  | 1 |  |  |  |  |  |  | 2 |  | g |
| WDG-048 |  |  |  |  |  |  |  |  |  |  |  |  | 1 |  | 1 |  |  |  |  | 2 |  | g |
| WDG-050 | 1 |  |  | 1 |  |  |  |  |  |  |  |  |  |  |  |  |  |  |  | 2 |  | g |
| WDG-053 | 1 |  |  |  |  |  |  |  |  |  |  |  |  |  |  |  | 1 |  |  | 2 | c |  |
| WDG-054 | 1 |  |  |  |  |  |  |  |  |  |  |  |  |  |  |  |  | 1 |  | 2 |  | g |
| WDG-055 |  |  |  |  |  |  |  |  |  |  |  |  |  | 1 |  |  |  |  | 1 | 2 |  | g |
| WDG-057 |  |  |  |  |  |  |  |  |  |  |  |  |  |  |  |  | 1 |  | 1 | 2 | c |  |
| WDG-059 | 1 |  |  |  |  |  |  | 1 |  |  |  |  |  |  |  |  |  |  |  | 2 |  | g |
| WDG-066 |  |  |  |  |  |  |  |  |  |  |  |  |  |  |  | 1 |  |  | 1 | 2 | c |  |
| WDG-069 | 1 |  |  |  |  |  |  |  |  |  |  |  |  |  |  |  |  | 1 |  | 2 |  | g |
| WDG-072 |  |  |  |  |  |  |  |  |  |  |  |  | 1 |  |  |  |  | 1 |  | 2 |  | g |
| WDG-077 | 1 |  |  |  |  | 1 |  |  |  |  |  |  |  |  |  |  |  |  |  | 2 |  | g |
| WDG-080 | 1 |  |  |  |  |  |  |  |  |  |  |  | 1 |  |  |  |  |  |  | 2 |  | g |
| WDG-092 |  | 1 |  |  |  | 1 |  |  |  |  |  |  |  |  |  |  |  |  |  | 2 |  | g |
| WDG-096 |  |  |  |  |  |  |  |  |  |  |  |  | 1 | 1 |  |  |  |  |  | 2 |  | g |
| WDG-103 |  |  |  |  |  |  |  |  |  |  |  |  | 1 | 1 |  |  |  |  |  | 2 |  | g |
| WDG-105 |  |  |  |  |  |  |  |  |  |  |  |  |  | 1 |  |  |  |  | 1 | 2 |  | g |
| WDG-111 |  |  |  |  |  |  |  |  |  |  |  |  |  |  |  |  | 1 |  | 1 | 2 | c |  |
| WDG-115 |  |  |  |  |  |  |  |  |  |  |  |  | 1 | 1 |  |  |  |  |  | 2 |  | g |
| WDG-118 |  |  |  |  |  |  |  |  |  |  |  |  | 1 | 1 |  |  |  |  |  | 2 |  | g |
| WDG-119 | 1 |  |  |  |  |  | 1 |  |  |  |  |  |  |  |  |  |  |  |  | 2 |  | g |
| WDG-135 | 1 |  |  |  |  |  |  |  |  |  |  |  |  |  |  |  |  | 1 |  | 2 |  | g |
| WDG-145 |  |  |  |  |  |  |  |  |  |  |  |  |  | 1 |  |  |  | 1 |  | 2 |  | g |
| WDG-157 |  |  |  |  |  |  |  |  |  |  |  |  |  |  |  | 1 |  |  | 1 | 2 |  | g |
| WDG-161 | 1 |  |  |  |  |  |  |  | 1 |  |  |  |  |  |  |  |  |  |  | 2 |  | g |
| WDG-001 |  |  |  |  |  |  |  |  |  |  |  |  | 1 |  |  |  |  |  |  | 1 |  | g |
| WDG-003 |  |  |  |  |  |  |  |  |  |  |  |  | 1 |  |  |  |  |  |  | 1 |  | g |
| WDG-013 |  |  |  |  |  |  |  |  |  |  |  |  |  | 1 |  |  |  |  |  | 1 |  | g |
| WDG-015 |  |  |  |  |  |  |  |  |  |  |  |  |  | 1 |  |  |  |  |  | 1 |  | g |
| WDG-019 |  |  |  |  |  |  |  |  |  |  |  |  | 1 |  |  |  |  |  |  | 1 |  | g |
| WDG-021 |  |  |  |  |  |  |  |  |  |  |  |  |  |  |  |  |  | 1 |  | 1 |  | g |
| WDG-023 |  |  |  |  |  |  | 1 |  |  |  |  |  |  |  |  |  |  |  |  | 1 |  | g |
| WDG-024 | 1 |  |  |  |  |  |  |  |  |  |  |  |  |  |  |  |  |  |  | 1 |  | g |
| WDG-030 |  |  |  |  |  |  |  |  |  |  |  |  |  |  |  |  |  |  | 1 | 1 |  | g |
| WDG-036 |  |  |  |  |  |  |  |  |  |  |  |  |  | 1 |  |  |  |  |  | 1 |  | g |
| WDG-038 |  |  |  |  |  |  |  |  |  |  |  |  |  |  | 1 |  |  |  |  | 1 |  | g |
| WDG-040 |  |  |  |  |  |  |  |  |  |  |  |  |  | 1 |  |  |  |  |  | 1 |  | g |
| WDG-041 |  |  |  |  |  |  |  |  |  |  |  |  |  |  |  |  |  | 1 |  | 1 |  | g |
| WDG-042 |  |  |  |  |  |  |  |  |  |  |  |  |  | 1 |  |  |  |  |  | 1 |  | g |
| WDG-044 |  |  |  |  |  |  |  |  |  |  |  |  | 1 |  |  |  |  |  |  | 1 |  | g |
| WDG-047 | 1 |  |  |  |  |  |  |  |  |  |  |  |  |  |  |  |  |  |  | 1 |  | g |
| WDG-049 |  |  |  |  |  |  |  |  |  |  |  |  |  | 1 |  |  |  |  |  | 1 |  | g |
| WDG-051 |  |  |  |  |  |  |  |  |  |  |  |  |  |  |  |  |  |  | 1 | 1 |  | g |
| WDG-058 |  |  |  |  |  |  |  |  |  |  |  |  |  | 1 |  |  |  |  |  | 1 |  | g |
| WDG-060 |  |  |  |  |  |  |  |  |  |  |  |  | 1 |  |  |  |  |  |  | 1 |  | g |
| WDG-064 |  |  |  |  |  |  |  |  |  |  |  |  | 1 |  |  |  |  |  |  | 1 |  | g |
| WDG-065 |  |  |  |  |  |  |  |  |  |  |  |  | 1 |  |  |  |  |  |  | 1 |  | g |
| WDG-074 | 1 |  |  |  |  |  |  |  |  |  |  |  |  |  |  |  |  |  |  | 1 |  | g |
| WDG-078 | 1 |  |  |  |  |  |  |  |  |  |  |  |  |  |  |  |  |  |  | 1 |  | g |
| WDG-088 | 1 |  |  |  |  |  |  |  |  |  |  |  |  |  |  |  |  |  |  | 1 |  | g |
| WDG-091 | 1 |  |  |  |  |  |  |  |  |  |  |  |  |  |  |  |  |  |  | 1 |  | g |
| WDG-097 |  |  |  |  |  |  |  |  |  |  |  |  |  |  |  |  |  |  | 1 | 1 |  | g |
| WDG-099 |  |  |  |  |  |  |  |  |  |  |  |  |  |  |  |  |  | 1 |  | 1 |  | g |
| WDG-102 |  |  |  |  |  |  |  |  |  |  |  |  | 1 |  |  |  |  |  |  | 1 |  | g |
| WDG-108 |  |  |  |  |  |  |  |  |  |  |  |  | 1 |  |  |  |  |  |  | 1 |  | g |
| WDG-109 |  |  |  |  |  |  |  |  |  |  |  |  |  |  |  |  | 1 |  |  | 1 | c |  |
| WDG-112 |  |  |  |  |  |  |  |  |  |  |  |  |  |  |  |  |  |  | 1 | 1 | c |  |
| WDG-113 |  |  |  |  |  |  |  |  |  |  |  |  |  |  |  |  |  | 1 |  | 1 |  | g |
| WDG-123 |  |  |  |  |  |  |  |  |  |  |  |  |  | 1 |  |  |  |  |  | 1 |  | g |
| WDG-125 |  |  |  |  |  |  |  |  |  |  |  |  |  | 1 |  |  |  |  |  | 1 |  | g |
| WDG-129 |  |  |  |  |  |  |  |  |  |  |  |  |  |  |  |  | 1 |  |  | 1 | c |  |
| WDG-131 |  |  |  |  |  |  |  |  |  |  |  |  | 1 |  |  |  |  |  |  | 1 | c |  |
| WDG-134 |  |  |  |  |  |  |  |  |  |  |  |  |  | 1 |  |  |  |  |  | 1 |  | g |
| WDG-136 |  |  |  |  |  |  |  |  |  |  |  |  |  |  |  |  | 1 |  |  | 1 |  | g |
| WDG-140 |  |  |  |  |  |  |  |  |  |  |  |  |  | 1 |  |  |  |  |  | 1 |  | g |
| WDG-149 |  |  |  |  |  |  |  |  |  |  |  |  |  |  | 1 |  |  |  |  | 1 |  | g |
| WDG-164 |  |  |  |  |  |  |  |  |  |  |  |  |  | 1 |  |  |  |  |  | 1 |  | g |
| **TOTAL** | 32 | 2 | 6 | 1 | 1 | 5 | 3 | 5 | 2 | 4 | 1 | 1 | 32 | 27 | 6 | 3 | 11 | 10 | 17 |  | 16 | 76 |
|  |  |  |  |  | 42 |  |  |  |  |  |  | 21 |  |  |  |  |  |  | 106 |  |  |  |
