# Supplementary material for: Adenovirus and Herpesvirus Diversity in Free-Ranging Great Apes in the Sangha Region of the Republic of Congo
Source: PLoS One. 2015 Mar 17;10(3):e0118543. doi: 10.1371/journal.pone.0118543 (PMC4362762; doi:10.1371/journal.pone.0118543)
Supplement: S2 Table — National Center for Biotechnology Information: http://www.blast.ncbi.nlm.nih.gov/Blast.cgi. (DOCX) [file pone.0118543.s006.docx]

S2 Table: Virus identification based on BLASTn

| **Virus subfamily/family** | **Identified Virus** | **% identity by BLASTn** | **Accession No.** |
| --- | --- | --- | --- |
| Gammaherpesvirinae | GgorLCV1 | 100.0 | GgorLCV1, AF290600 |
|  | GgorLCV2 | 99.4 | GgorLCV2, AY129395 |
|  | PtroLCV1 | 99.4 | PtroLCV1, AY166457 |
|  | GgorRHV1 | 99.4 | GgorRHV1, AY177144 |
|  | PtroRHV1 | 100.0 | PtroRHV1, AF250880 |
| Betaherpesvirinae | GgorCMV1.1 Group 1 | 100.0 | GgorCMV1.1, FJ538492 |
|  | GgorCMV1.1 Group 2 | **95.4** | GgorCMV1.1, FJ538492 |
|  | GgorCMV 2.1 | 98.0 | GgorCMV2.1, FJ538490 |
|  | GgorCMV2.2 Group 1 | 99.5 | GgorCMV2.2, FJ538491 |
|  | GgorCMV2.2 Group 2 | **96.9** | GgorCMV2.2, FJ538492 |
|  | PtroCMV1.1 | 98.4 | PtroCMV1.1, FJ538485 |
|  | PtroCMV2.1 | 98.5 | PtroCMV2.1, FJ538487 |
| Adenoviridae | SAdVGroupOKNP (WDG65) | **89.4** | SAdV28.1/41.2/41.1, FJ025914 ; FJ025927 ; FJ025913 |
|  | SAdVGroup27.1/28.2/29/46/47 | 99.0 | SAdV27.1/46/47, FJ025909; FJ025930; FJ025929 |
|  | SAdVGroup27.2/28.1/32/41.1/41.2 | 99.5 | SAdV28.1/41.2/41.1, FJ025914 ; FJ025927 ; FJ025913 |
|  | SAdVGroup35.1/35.2 | 99.5 | SAdV35.1 and 35.2, FJ025912 ; FJ025910 |
|  | SAdV31.2 | 100.0 | SAdV31.2, FJ025904 |
|  | SAdVGroup43/45 | 100.0 | SAdV43 and 45, FJ025900; FJ025901 |
|  | SAdVGroup39/25/26 | 99.0 | SAdV39/25/26, FJ025924;JN254802; FJ025923 |
